# Respiratory Syncytial Virus Infection: Enhanced Reactive Oxygen Species Production and Weakened Antioxidant Defenses Worsen Disease Severity

**DOI:** 10.3390/v18101146

**Published:** 2026-10-15

**Authors:** Oliviero Sacco, Paola Borgia, Giorgia M. Rossi, Angelo Florio, Giovanni A. Rossi

**Affiliations:** 1 Pediatric Pulmonology and Respiratory Endoscopy Unit, Istituto di Ricovero e Cura a Carattere Scientifico (IRCCS) Istituto Giannina Gaslini, 16147 Genoa, Italy; olivierosacco@gaslini.org (O.S.); paolaborgia@gaslini.org (P.B.); angeloflorio@gaslini.org (A.F.); 2 Department of Internal Medicine, Università di Genova, 16132 Genova, Italy; giorgia.m.rossi93@gmail.com

**Keywords:** viral infections, bronchiolitis, oxidative stress, airway epithelial cells, innate immune response, innate lymphoid cell2

## Abstract

Respiratory syncytial virus (RSV) is a leading cause of severe respiratory infections in infants and young children globally. The severity of RSV-associated diseases is often ascribed to a dysregulated immune response, characterized by increased secretion of proinflammatory proteins and ineffective T helper type 2 (Th2) cell activity. The host’s primary defense against RSV relies on a well-coordinated innate immune response initiated by resident airway epithelial cells, the first target of RSV infection. These cells respond to viral invasion by generating reactive oxygen species (ROS), activating the production of Type I and III interferons, and releasing proinflammatory cytokines and chemokines. Moderate levels of ROS act as key molecules that regulate antiviral signaling, enhancing the host’s ability to recognize and eliminate invading pathogens. However, severe RSV infection disrupts cellular homeostasis triggering excessive ROS production, while simultaneously weakening the host’s antioxidant defenses. This redox imbalance subverts the activity of key cellular defensive mechanisms, ultimately increasing viral replication, and worsening RSV infection severity. Modulation of oxidative stress may offer an innovative approach in managing the progression and outcome of RSV infection.

## 1. Introduction

Respiratory syncytial virus (RSV) is a major viral pathogen responsible for substantial morbidity and mortality among infants and young children [1,2]. RSV infection is a complex, multistage process, and disease severity is often ascribed to a dysregulated immune response characterized by increased secretion of proinflammatory proteins and ineffective T helper type 2 (Th2) cell activity [3,4,5]. Although new vaccines and monoclonal antibodies are available for specific patient groups [6], a clearer understanding of the cellular factors and signaling events that regulate RSV infection could support the development of affordable, effective therapies. Early severe infection is characterized by high viral replication, extensive uncontrolled inflammation, epithelial injury, interstitial mononuclear infiltrates, and intra-alveolar edema [7]. Airway lumens become obstructed by debris composed of desquamated epithelial cells, macrophages, neutrophils, and degranulated eosinophils mixed with fibrin and mucin [7,8]. This widespread airway damage is largely attributed to an early, dysregulated innate immune response initiated by airway epithelial cells, the primary targets of RSV infection and replication [9,10]. Pattern recognition receptors (PRRs) detect RSV proteins, RNA, viral transcripts, and replication intermediates, triggering early signaling in airway epithelial cells and the production of reactive oxygen species (ROS) [11,12,13]. This is followed by the activation of multiple transcription factors, including interferon-gamma (IFN-γ), and of signaling pathways that promote cytokine production and an antiviral response [10]. The infection then reaches the lower respiratory tract through the release of viral progeny by budding and through cell-to-cell spread along intracytoplasmic bridges. Effective control of ROS by antioxidant systems enhances innate immunity and strengthens antiviral defenses [12,13]. In severe infection, however, excessive ROS production is accompanied by broad suppression of antioxidant enzyme expression, resulting in redox imbalance, lung inflammation, and tissue injury [13,14,15]. Oxidative stress also weakens several defense mechanisms, thereby promoting rather than limiting viral replication, driving Th2-type inflammation, and aggravating lung pathology.

## 2. ROS Production and Oxidative Stress

ROS are byproducts of the partial reduction of molecular oxygen (O_2_). In healthy cells, their concentrations remain low, allowing them to regulate processes such as proliferation, differentiation, migration, and necrosis [16,17]. ROS originates from several cellular sites, including plasma membrane, mitochondria, endoplasmic reticulum (ER), phagosomes, and endosomes. During viral infections, however, the mitochondrial electron transport chain (ETC) is the primary source of ROS, being a key component of cellular respiration and energy metabolism [18,19]. The ETC comprises four protein complexes (Complexes I–IV) and receives electrons from nicotinamide adenine dinucleotide phosphate (NADPH) oxidases (NOX) [19,20]. Humans express seven NOX isoforms—NOX1–5 and DUOX1/2—which differ in tissue distribution, subunit composition, and localization. In mitochondria, NOX2 transfers electrons from NADPH to O_2_, generating superoxide anions (O_2_^−^), which are then converted by superoxide dismutase (SOD) into hydrogen peroxide (H_2_O_2_) [21]. Catalase (CAT) and glutathione peroxidases (GPxs) primarily convert H_2_O_2_ into water (H_2_O). Under oxidative stress, however, H_2_O_2_ reacts with ferric iron (Fe^3+^) or copper (Cu^2+^) ions through the Fenton reaction, producing highly reactive, short-lived hydroxyl radicals (HO·) [22]. Smaller amounts of ROS are also generated by flavin-centered oxidases in microsomes and peroxisomes, oxidative protein folding in the ER, and by fatty-acid β-oxidation in peroxisomes [23,24,25]. Although ROS production is tightly controlled in quiescent cells, hypoxia, inflammation, and infection can increase it to levels that overwhelm cellular protective mechanisms [26,27].

## 3. Antioxidant Defense Mechanisms

**Figure 1:**
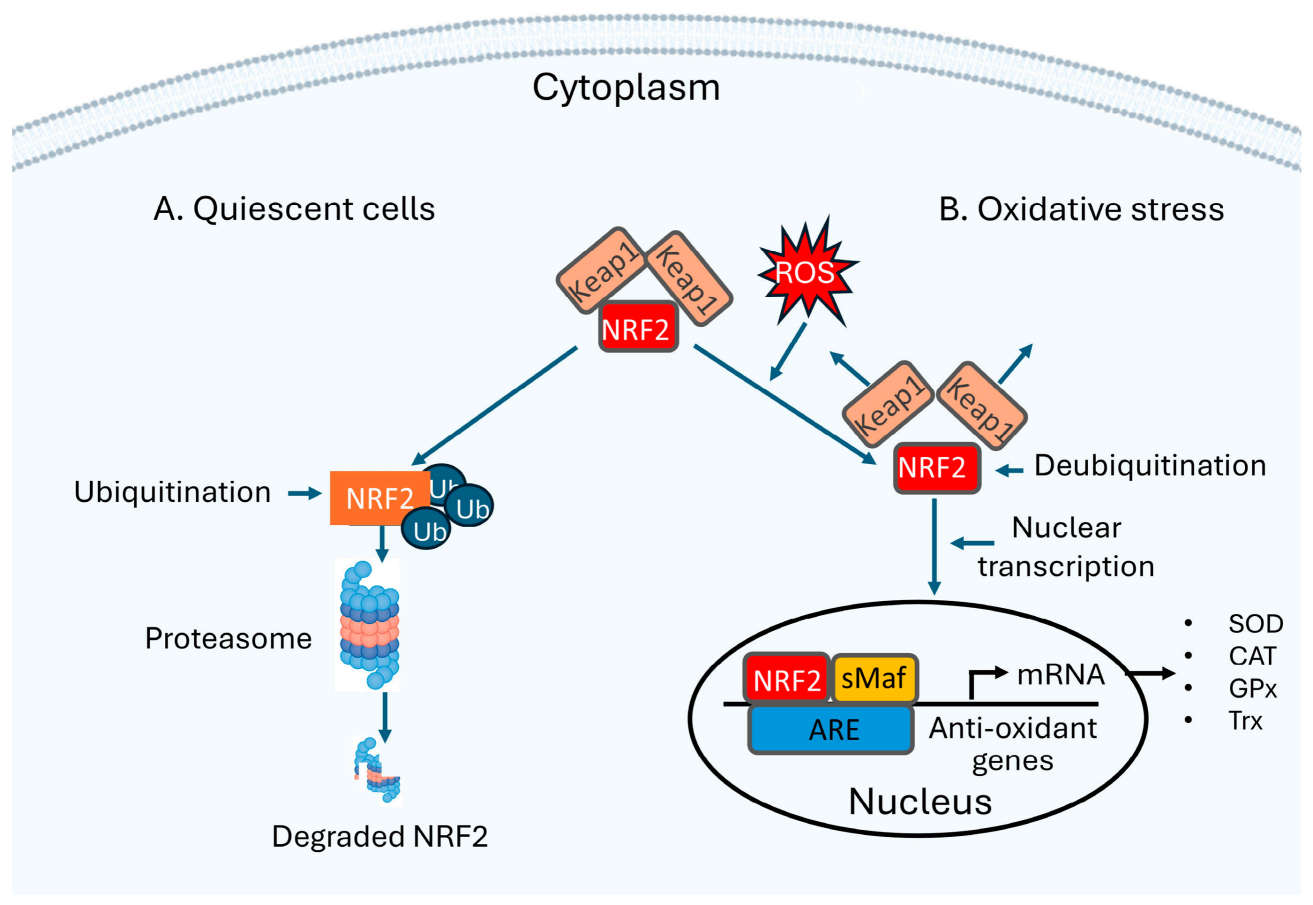
NRF2 pathway regulation. A. In resting cells, KEAP1 binds NRF2, promoting its ubiquitination and proteasomal degradation. B. Under oxidative stress, NRF2 dissociates from KEAP1, translocates to the nucleus, and forms a heterodimer with small Maf proteins. This complex binds antioxidant response elements (AREs) in DNA and activates antioxidant gene transcription.

Cells preserve redox homeostasis through enzymatic and nonenzymatic antioxidant systems. Key enzymatic antioxidants include SOD isoforms 1–3, CAT, glutathione (GSH), GPx, glutathione S-transferases (GSTs), peroxiredoxins (Prdx), and thioredoxin reductases (Trx) [13,14]. GSH is a major ROS scavenger, and the ratio of GSH to its oxidized form, GSSG, indicates the level of oxidative stress [13,14]. A high GSH/GSSG ratio reflects a healthy redox balance, whereas a low ratio signal reflects increased oxidative stress and weakened antioxidant defenses. Nonenzymatic antioxidants, including vitamins A, C, and E and minerals such as selenium and zinc, directly neutralize ROS and support antioxidant enzyme activity [13,14]. Moreover, nuclear factor erythroid 2-related factor 2 (NRF2) regulates the expression of antioxidant enzymes and cytoprotective proteins, coordinating the cellular response to oxidative stress (Figure 1) [28]. In resting cells, NRF2 binds Kelch-like ECH-associated protein (KEAP1), which promotes its degradation. Under oxidative stress, NRF2 escapes KEAP1-mediated suppression, translocates to the nucleus, forms a heterodimer with small Maf proteins, and binds antioxidant response elements (AREs) in DNA to induce antioxidant gene transcription [28,29]. NRF2 activation limits redox-induced cellular injury and may reduce viral replication and inflammation during infection [30]. However, some viruses, including RSV, suppress NRF2 production to promote their replication [31].

## 4. ROS Generation in RSV Infection

**Figure 2:**
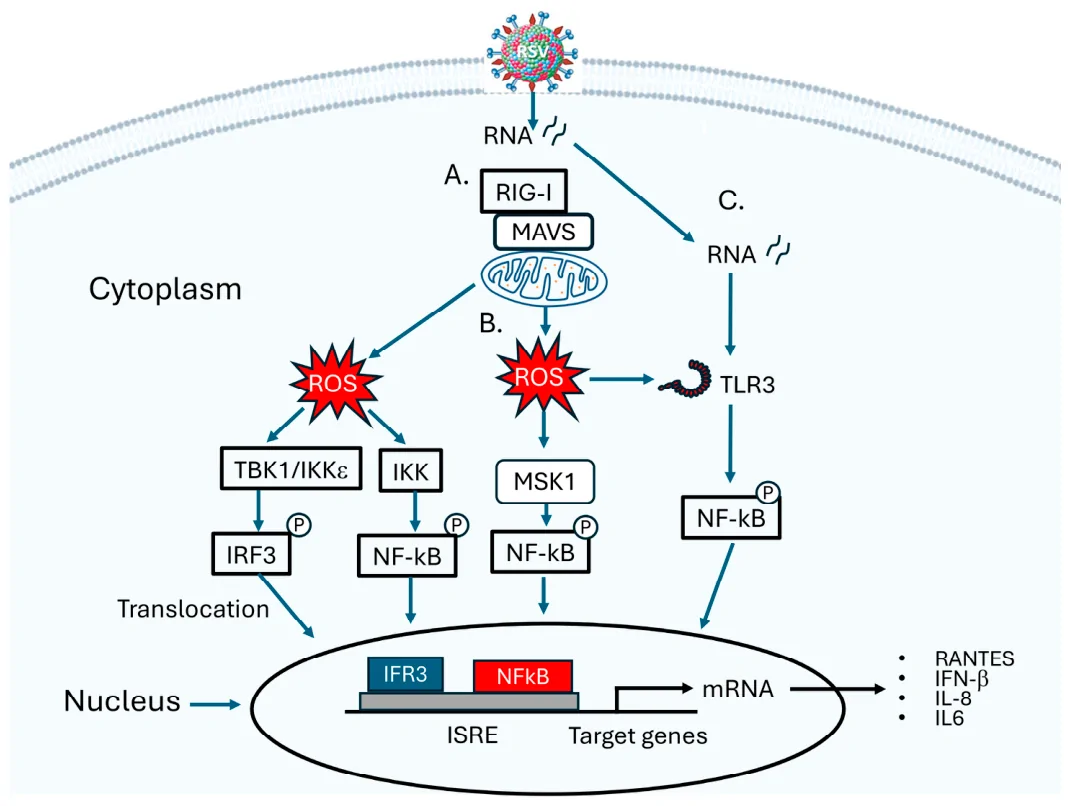
ROS-driven antiviral cytokine production in airway epithelial cells infected with RSV. (**A**). RSV RNA sensing activates the RIG-I–MAVS pathway, rapidly generating ROS and stimulating IKK kinase complexes. These complexes phosphorylate IRF3 and NF-κB and promote their nuclear translocation. IRF3 and NF-κB then bind interferon-stimulated response elements (ISREs), inducing antiviral cytokine and chemokine expression. (**B**). ROS-dependent activation of MSK1 provides an additional upstream mechanism regulating RSV-induced NF-κB phosphorylation. (**C**). ROS production also enhances TLR3-mediated RSV RNA sensing, leading to NF-κB activation.

The mechanisms underlying ROS generation during RSV infection have been investigated in cell cultures and experimental mouse models [11,12]. In airway epithelial cells, retinoic acid-inducible gene I (RIG-I) detects RSV RNA and viral replication intermediates, initiating early signaling [32,33]. At the mitochondrial surface, RIG-I forms a complex with mitochondrial antiviral signaling protein (MAVS) (Figure 2A) [33]. Relocation of the RIG-I–MAVS complex toward the microtubule-organizing center (MTOC) causes perinuclear mitochondrial clustering and an asymmetric distribution around the MTOC [34]. This reorganization disrupts the mitochondrial membrane potential (Δψ_m_) and increases mitochondrial ROS production, thereby promoting RSV replication and lung inflammation [34]. RIG-I–MAVS-dependent ROS generation is required to activate interferon regulatory factor 3 (IRF3) and NF-κB and to induce antiviral cytokine and chemokine expression [35,36] (Figure 2A). In RSV-infected cells, ROS production follows a biphasic pattern [37]: an initial peak occurs 2–3 h after RSV RNA internalization and recognition by cytosolic sensors, followed by a second peak 6–12 h later during early viral replication. Proinflammatory cytokine and chemokine production peaks at 6–12 h post-infection and is inhibited by antioxidants. ROS-dependent activation of mitogen- and stress-activated kinase 1 (MSK1) is another upstream mechanism regulating RSV-induced NF-κB phosphorylation (Figure 2B) [11,35]. Oxidative stress also enhances TLR3 activation, which further regulates NF-κB phosphorylation (Figure 2C) [35,38]. ROS can also be generated by ER stress, as unfolded RSV glycoproteins activate UPR, leading to ER Ca^2+^ release, mitochondrial overload, increased ROS, and cytochrome C release. ROS may also arise from ER stress. Accumulation of unfolded or misfolded RSV F, G, and SH glycoproteins in the ER lumen activates the unfolded protein response (UPR). If normal cellular function is not restored, the UPR promotes ER Ca^2+^ release and mitochondrial Ca^2+^ uptake, causing mitochondrial Ca^2+^ overload, increased ROS production, greater transition pore permeability, and cytochrome c release [39,40]. Massive ROS generation induces chemokine release, recruiting and activating neutrophils that further produce ROS, overwhelming antioxidant defenses [13,41,42,43].

## 5. Reduction of Antioxidant Activity in RSV Infection

**Figure 3:**
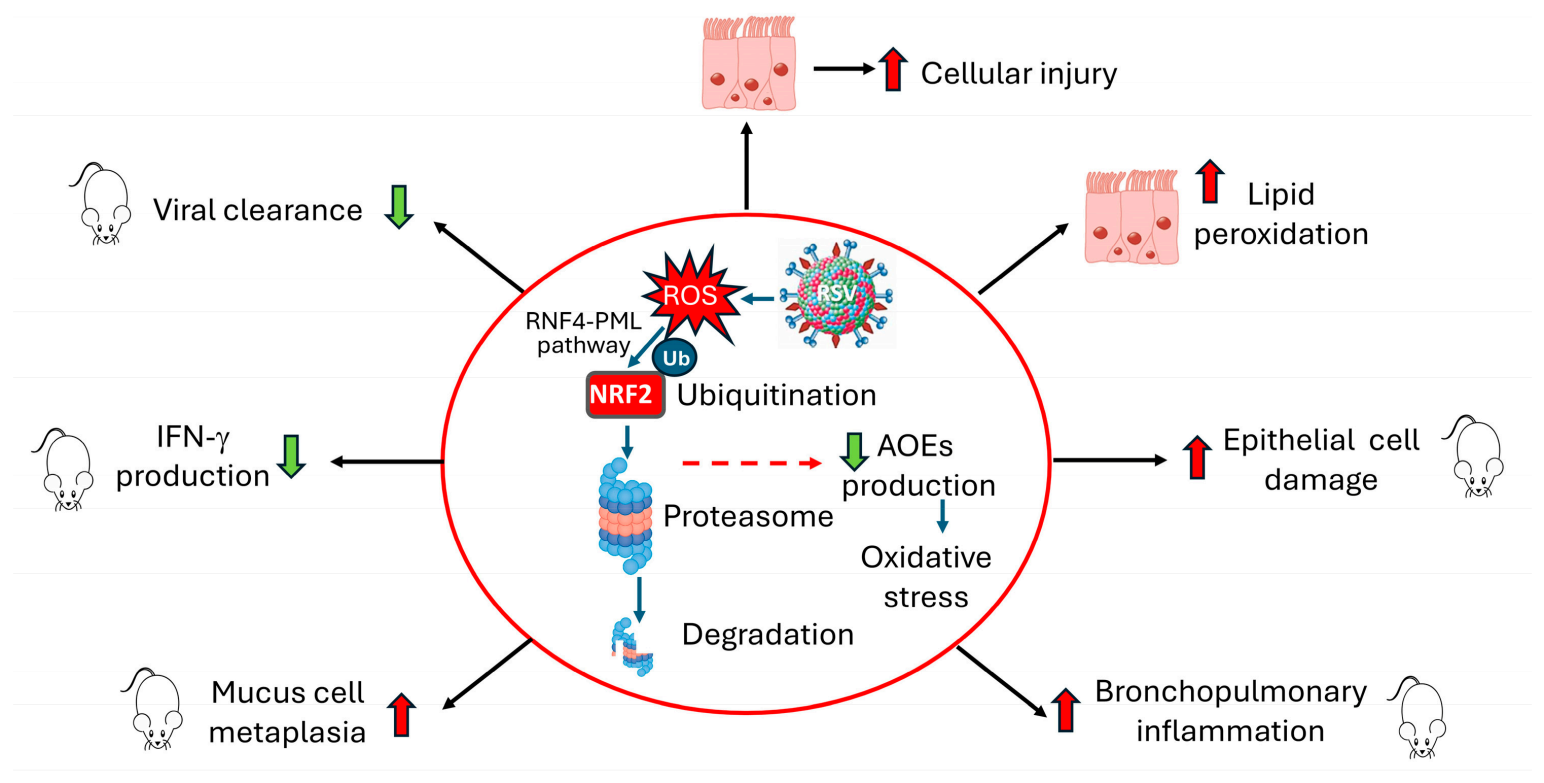
Excess ROS activates the RNF4–PML pathway, accelerating proteasomal degradation of NRF2 and lowering the expression of several antioxidant enzymes. This oxidative imbalance damages airway epithelial cells and worsens RSV infection.

In RSV infection, oxidative stress is further promoted by proteasomal degradation of the transcription factor NRF2 (Figure 3) [15]. ROS exposure activates the RNF4–PML (RING finger protein 4–promyelocytic leukemia) pathway, a key cellular mechanism exploited by RSV to reduce antioxidant production [15]. In cultured airway epithelial cells, RSV progressively decreases nuclear and total cellular NRF2 levels, downregulating several antioxidant enzymes (AOEs) and thereby promoting lipid peroxidation and oxidative stress [15,31]. Compared with RSV-infected wild-type (NRF2^+/+^) mice, NRF2-deficient (NRF2^−/−^) mice show significantly greater inflammation, epithelial damage, mucus cell metaplasia, and nasal injury [44]. They also exhibit increased ROS production, reduced AOE and IFN-γ levels, and impaired viral clearance. Reduced NRF2 expression and AOE levels have likewise been detected in bronchoalveolar lavage (BAL) fluid from RSV-infected mice and in nasopharyngeal secretions from infants with severe bronchiolitis, in whom markers of oxidative damage correlate with clinical disease severity [31].

## 6. Host Defense Mechanisms in RSV Infection

**Figure 4:**
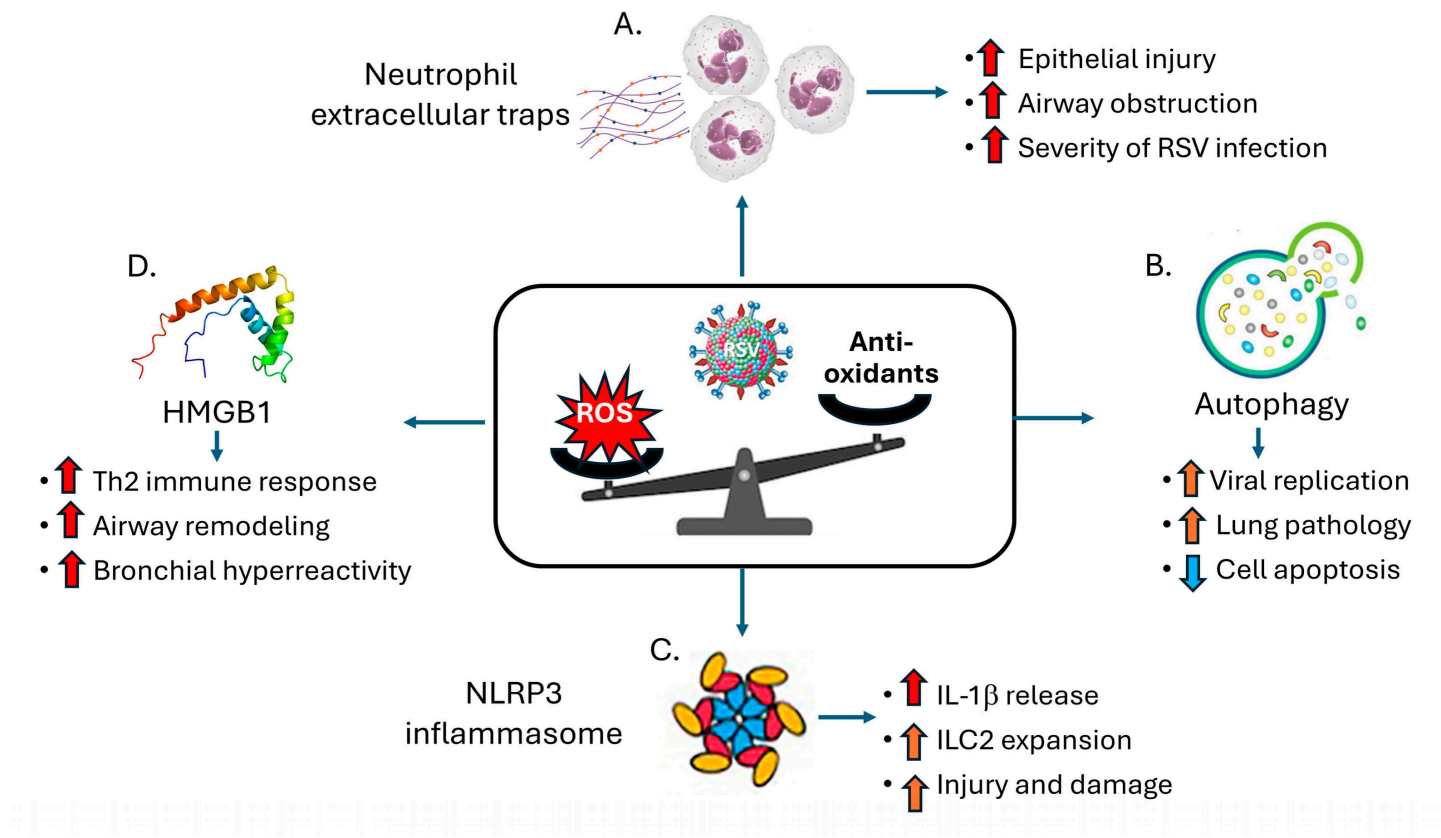
During RSV infection, redox imbalance impairs several cellular defense mechanisms: (**A**) neutrophil extracellular traps; (**B**) autophagy; (**C**) the NLRP3 inflammasome; and (**D**) high-mobility group box 1 (HMGB1).

During RSV infection, excessive ROS production and impaired antioxidant defenses disrupt key host-defense mechanisms, including neutrophil extracellular trap formation, autophagic flux, Nod-like receptor protein 3 (NLRP3) inflammasome activation, and high-mobility group box 1 release. This dysfunction promotes RSV replication and lung inflammation. In healthy cells, these mechanisms are protective, as described in the following sections [13] (Figure 4).

### 6.1. Neutrophil Extracellular Traps Formation

A hallmark of RSV infection is the marked recruitment of neutrophils to affected tissues [42]. As first-line responders, neutrophils activate multiple defense mechanisms, including ROS production, degranulation, and the release of web-like neutrophil extracellular traps (NETs) through a form of programmed cell death known as NETosis [45]. This is a process largely driven by ROS bursts, produced by mitochondrial NADPH oxidase. NETs are composed of depolymerized chromatin filaments (DNA and histones), heavily decorated by the antimicrobial granular proteases, myeloperoxidase (MPO), neutrophil elastase (NE), and cathepsin G that, following NETosis, are released from cells [46]. NETs exhibit defensive properties in that their sticky filaments can trap and deactivate free virions and through antimicrobial peptides and enzymes stored in neutrophil granules that target and neutralize invading pathogens [47]. However, NETs release can also result in collateral injury to host parenchymal cells, with the extent of damage depending on the context and the degree of NET formation [48]. Studies in human neutrophils showed that RSV induced the classical ROS-dependent NETosis, with RSV being trapped in DNA lattices, coated with NE and MPO [49]. Moreover, RSV infection of airway epithelial cells triggered NET-DNA release by neutrophils, indicating that neutrophils can identify RSV-infected cells and respond to them by releasing NETs [49]. Early research showed that co-incubation of HEp-2 epithelial cells with NET, at concentrations ≥ 2 μg/mL, resulted in statistically significant cell death, with histones contributing to damage [50]. Conversely, when NETs, at concentrations below 16 μg/mL, were co-incubated with RSV particles for 1 h, protection of HEp-2 cells from viral infection was demonstrated. However, cell protection decreased with increasing concentrations of NETs and resulted in cell damage [50]. A more recent investigation, from the same research group, demonstrated that neither the pre incubation with NETs (0.5–16 μg/mL), 2 h prior to RSV infection, nor the NETs addition, 2 h after infection, conferred any protective effect [51]. Excessive NET formation is linked to disease severity, as highlighted in mice deficient in the receptor for IFN-λ, a cytokine known to suppress neutrophilic inflammation [52]. As compared to wild-type animals, 28R-deficient mice infected with a rodent-specific pneumovirus exhibited increased ROS production and NETosis, resulting in early neutrophilic inflammation and epithelial injury (Figure 4A) [52]. In children with RSV-induced respiratory failure, increased NETosis was observed in bronchoalveolar lavage fluid (BALF), correlating with disease severity [53]. Similar findings were seen in animal models, where neutrophilic inflammation and NETs release caused airway obstruction, both in small airways and larger bronchi [53]. Treatment of dornase alfa, a therapeutic agent that breaks down extracellular DNA, resulted in a strong reduction in NETs-induced airway obstruction [53].

### 6.2. Autophagy Flux Induction and Apoptosis Inhibition

Autophagy is a cellular recycling process that degrades unused proteins and cellular debris and regulates multiple cell functions [54]. During viral infection, it supports host defense by eliminating viral proteins and presenting them to antigen-presenting cells, thereby promoting Th1 responses [55,56]. However, some viruses manipulate autophagy to facilitate replication in airway epithelial cells, depending on the virus, host cell type, and disease severity [56,57]. RSV research has mainly examined autophagy in antigen presentation and infection resolution [58,59], but excessive ROS production may reverse its protective effects. In HEp-2 cells, RSV-induced ROS activated the AMP-activated protein kinase–mammalian target of rapamycin (AMPK–mTOR) pathway, promoting autophagosome formation, fusion with lysosomes, and degradation and release of protein contents [60]. Autophagy increased expression of RSV structural and non-structural proteins and raised viral loads (Figure 4B). Treatment with the ROS scavenger N-acetyl-L-cysteine or the AMPK–mTOR inhibitor 3-MA reduced RSV gene expression and viral load, with N-acetyl-L-cysteine acting earlier and more strongly. In lung homogenates from RSV-infected BALB/c mice, autophagosome activity appeared 3 days after infection and was associated with increased NS1 and NS2 gene expression and viral titers [59]. These effects were markedly reduced by 3-MA, which also decreased HEp-2 cell viability and promoted apoptosis [60]. Apoptosis limits viral replication and prevents the release of progeny virions and proinflammatory mediators [61]. In A549 cells, NS1 and NS2 were expressed early after RSV infection, whereas apoptosis occurred much later [62]. Their expression coincided with early activation or expression of anti-apoptotic factors, including NF-κB, glycogen synthase kinase, and phosphorylated AKT. Silencing NS proteins with specific small interfering RNAs (siRNAs) abolished this anti-apoptotic effect and triggered early cell death [62]. Similarly, in A549 epithelial cells transfected with an NS1-Flag plasmid, AMPK–mTOR-dependent autophagy suppressed inflammatory cytokine production and apoptosis [63].

### 6.3. NLRP3 Inflammasome Activation

The NLRP3 inflammasome is an innate immune multiprotein complex that detects pathogen- and damage-associated molecular patterns [13,64]. Once activated, it triggers caspase-1, which matures the proinflammatory cytokines IL-1β and IL-18 and cleaves Gasdermin D (GSDMD) [64,65]. During viral infection, IL-1β recruits neutrophils to support pathogen clearance, while IL-1β and IL-18 promote broader immune responses [66]. GSDMD mediates pyroptosis, a highly inflammatory form of programmed cell death marked by cell swelling, plasma membrane rupture, and IL-1β and IL-18 release [65]. Although essential for host defense, the NLRP3 inflammasome can be exploited by some viruses to enhance replication [67], and ROS are among its main activators [12]. In HEK293 cells and mouse bone marrow-derived macrophages, RSV-induced ROS activated NLRP3-associated caspase-1, converting pro-IL-1β and pro-IL-18 into their active forms and driving severe inflammation and tissue injury (Figure 4C) [68]. In neonatal monocytes, RSV increased IL-1β transcripts and protein release within 2 h of exposure; this response was sustained by IL-1β-converting enzyme and elevated interferon regulatory factor 1 expression [69]. In vivo, excessive NLRP3 activation and IL-1β production were strongly associated with severe Th2-type lung inflammation and injury. Tracheal secretions from infants with severe RSV infection contained markedly elevated IL-1β and RANTES, a proinflammatory chemokine involved in immune regulation and closely associated with Th2-driven allergic inflammation and eosinophilic disease [37,70]. In a neonatal mouse model, IL-1β increased pulmonary IL-33 expression by promoting proliferation of IL-33-positive lung epithelial stem/progenitor cells. This rise was accompanied by substantial lung infiltration of type 2 innate lymphoid cells (ILC2), increased airway mucus production, and marked methacholine hyperresponsiveness [70]. Complementary neonatal mouse studies similarly showed that early IL-1β release during RSV infection increased airway IL-33 expression and promoted a Th2-biased response upon reinfection [71].

### 6.4. High Mobility Group Box 1 Release

High-mobility group box 1 (HMGB1) is a damage-associated molecular pattern with multiple cellular functions [72]. It acts as an alarmin with a dual role: in healthy cells, HMGB1 supports essential nuclear processes, including DNA stabilization and transcriptional regulation; in response to infection or tissue damage, it contributes to host defense and immune surveillance [72,73]. However, oxidative stress can dysregulate HMGB1 and promote severe cellular and tissue injury [74,75]. Studies in airway epithelial cells and Fischer 344 rat pups showed that RSV markedly increases HMGB1 protein levels and that ROS-dependent signaling is required for its production and release [76]. In IRF7^−/−^ mice with PVM-induced viral bronchiolitis, increased epithelial and mesenchymal HMGB1 expression was associated with more IL-13-producing ILC2s and expansion of the airway smooth muscle (ASM) layer (Figure 4D) [77]. HMGB1 neutralization eliminated lung ILC2s and ASM growth in vivo and inhibited ILC2-mediated ASM cell proliferation in co-culture [77]. BALB/c mice intranasally infected with RSV also showed significantly increased HMGB1 levels in lung tissue and bronchoalveolar lavage fluid (BALF) [78]. HMGB1 was mainly localized to low columnar and cuboidal bronchiolar epithelial cells in the small airways and was associated with higher concentrations of the Th2 cytokines IL-4, IL-5, and IL-13. Anti-HMGB1 treatment significantly reduced clinical severity scores, BALF Th2 cytokine concentrations, peribronchiolar inflammatory-cell infiltration, and methacholine-induced airway hyperresponsiveness [78]. HMGB1 levels were also markedly elevated in nasopharyngeal aspirates from hospitalized infants with RSV bronchiolitis and correlated significantly with clinical severity and blood monocyte and lymphocyte counts [78].

## 7. RSV Infection, ILC2 Activation and Th2-Type Immune Response

**Figure 5:**
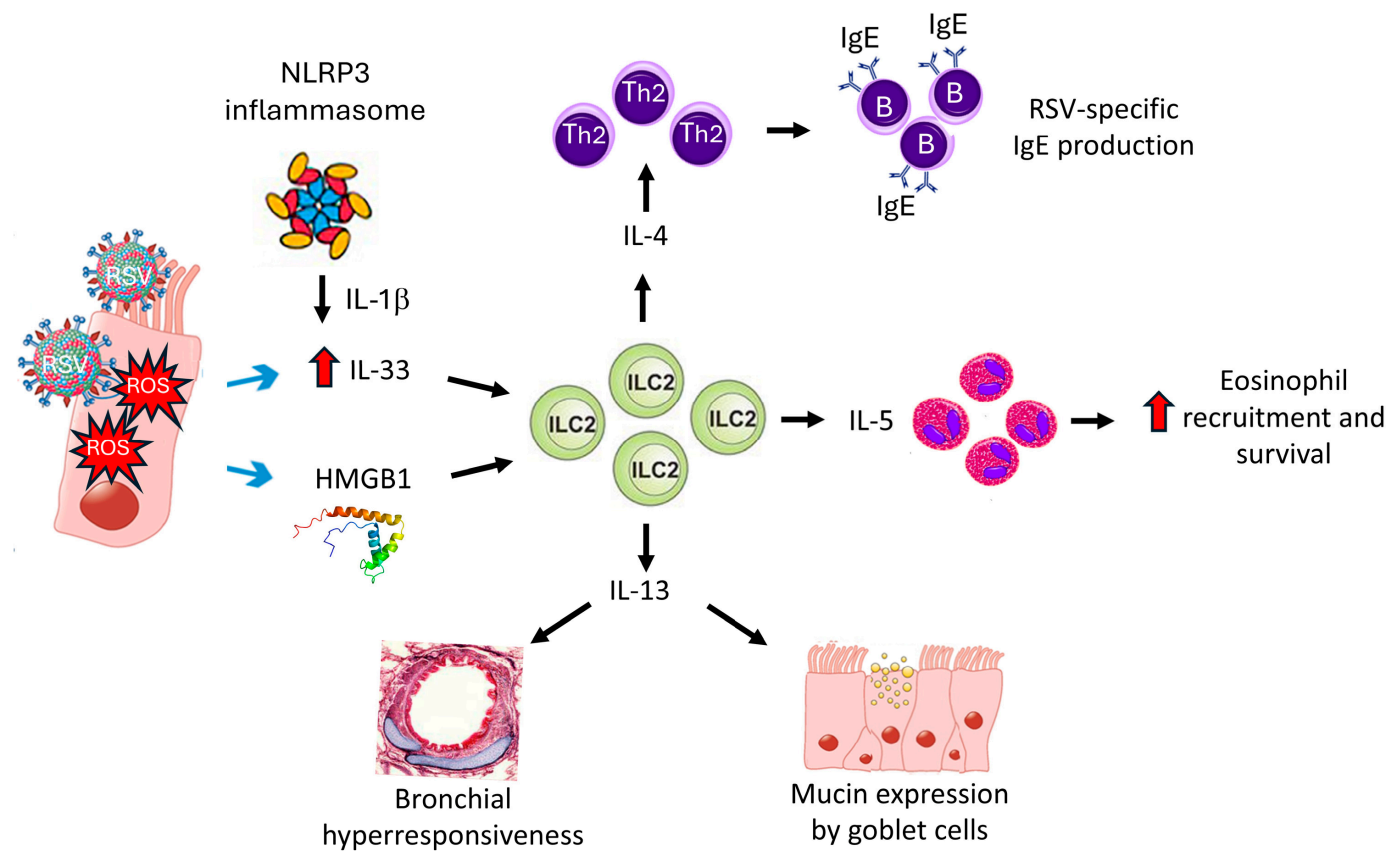
Crosstalk among RSV-infected airway epithelial cells, the NLRP3 inflammasome, and HMGB1 drives group 2 innate lymphoid cell (ILC2) proliferation and promotes a type 2 helper T-cell (Th2) inflammatory response.

NLRP3 inflammasome and HMGB1 activation in RSV infection upregulates ILC2 proliferation in lung tissue. ILC2, a population of innate cells of lymphoid origin, are known drivers of strong Th2 responses, independently from the involvement of Th2 cells [79,80]. Unlike T cells or B cells, ILC2 do not express antigen receptors and are activated by, cytokines called “alarmins”, released by airway epithelial cells in response to viral infections or allergen exposure [80]. ILC2 release high amounts of the Th2 cytokines IL-4, IL-5 and IL-13 (Figure 5) [81]. IL-4 drives Th2 cell differentiation and promotes B cell antibody class switch to IgE producing plasma cells. IL-5 is involved in eosinophils recruitment and enhanced survival. IL-13 induces airway hyperresponsiveness and increases mucin expression in airway goblet cells (Figure 5). Following RSV bronchiolitis infancy, long-term epithelial progenitors or persistent epigenetic modifications of airway epithelial cells may play a pathogenetic role in in the induction of type 2 programming in response to RSV infection and in increasing susceptibility to obstructive lung diseases in later life [80,81]. ILC2 activation can be involved in the promotion of the Th2-biased response described in the early phase of RSV infection. In a report from Southampton University, Th1/Th2 cytokine balance was evaluated in nasal lavage samples obtained from infants with RSV-induced upper respiratory tract infection [82]. Infants who subsequently developed acute bronchiolitis had elevated IL-4/INF-γ ratios on Days 1–2 and Days 5–7 of illness. In these infants, elevated mRNA IL-4/INF-γ ratios were also found in the supernatants of stimulated peripheral blood mononuclear cell cultures. Since there was no difference in the initial RSV load between the two infant groups, excessive Th2 immune responses may have had a pathogenetic role in the outcome of the infection [82]. Clinical studies have also tried to characterize the “atopic-like condition” observed in the early phase of severe RSV bronchiolitis. High levels of RSV-specific IgE and IgG4 were found in serum and in nasopharyngeal samples collected in RSV-infected infants, associated with eosinophilic inflammation and with elevated histamine concentrations and correlated with disease severity [83,84,85]. Although several studies suggest that infants with severe RSV bronchiolitis are at increased risk of recurrent wheezing and asthma later in childhood, the evidence linking RSV to allergic sensitization remains inconclusive. Clinical findings have been inconsistent, with some studies supporting this association and others failing to confirm it [81,86]. The long-term features of recurrent respiratory disorders in pediatric populations may reflect a complex interaction among genetic susceptibility, environmental exposures, and RSV-induced changes in immune function [81,86].

## 8. Therapeutic Potential of Antioxidants in RSV Infection

Experimental studies in airway epithelial cells and animal models indicate that targeting redox imbalance may protect against RSV infection [87]. Pretreating RSV-infected cells with antioxidants such as butylated hydroxyanisole (BHA) and other compounds suppressed proinflammatory chemokine production and viral replication, thereby reducing inflammation and tissue damage [37]. In RSV-infected mice, oral BHA or the mitochondria-targeted antioxidant mitoquinone mesylate (MitoQ) reduced viral replication, inflammatory-cell infiltration, bronchial hyperresponsiveness, and clinical symptoms [34,87]. Aerosolized recombinant superoxide dismutase (rhuSOD) also lowered pulmonary RSV titers in infected cotton rats [88]. Other antioxidants, including N-acetyl-L-cysteine (NAC), melatonin, and resveratrol, have shown therapeutic effects. In RSV-infected epithelial cells, NAC inhibited viral replication, viral gene expression, cytokine release, and mucin synthesis while improving cell survival [60,89]. Melatonin scavenges free radicals and enhances antioxidant enzyme activity [90]. In mice, oral melatonin reversed RSV-induced increases in nitric oxide (NO), malondialdehyde (MDA), and hydroxyl radicals (HO·), restored lung GSH and SOD activity, and reduced serum proinflammatory cytokine levels [91]. Resveratrol has antiviral and anti-inflammatory effects, but its poor water solubility and low oral bioavailability limit its use [92]. Nanotechnology can convert insoluble resveratrol (Res) into highly soluble nanoparticles (Res NPs) suitable for nebulization, prolonging pulmonary residence time [92]. In RSV-infected BALB/c mice, nebulized Res NPs reduced viral load in bronchoalveolar lavage fluid (BALF), attenuated bronchial constriction, inhibited IL-6 and TNF-α expression, and reduced lung pathology [93]. Because conventional antioxidants have provided limited clinical benefits, regulating RSV-induced changes in cellular metabolism may represent a new therapeutic strategy.

## 9. Conclusions

Excessive ROS production and the resulting oxidative stress likely initiate and amplify the lung injury and inflammation associated with severe RSV infection. Experimental evidence suggests that targeting ROS may offer therapeutic benefits; however, conventional antioxidants have shown limited clinical efficacy because of their nonspecific effects and poor bioavailability. Novel pharmacological agents and improved delivery strategies may overcome these limitations [94]. Preventing RSV-induced metabolic reprogramming, which supports viral replication, may also represent a promising therapeutic approach [95]. Future studies could use lung organoid cultures to investigate how metabolic alterations, oxidative stress, and disease mechanisms interact during RSV infection [96]. These miniaturized, simplified three-dimensional (3D) tissue cultures derived from stem cells that mimic the structural and functional complexity of real organs, provide useful human-cell-based platforms for studying the pathogenesis of diverse diseases, including respiratory viral infections [97,98].

## Data Availability

No new data were created, because data is unavailable due to privacy or ethical restrictions.

## Abbreviations

AMPKMTOR: AMP-activated protein kinase/mammalian target of rapamycin AOEs: Antioxidant enzymes; ARE: antioxidant response elements; BHA: butylated hydroxyanisole; CAT: Catalase; ER: Endoplasmic reticulum; RTC: electron transport chain, GSH: Glutathione; GSSG: Oxidized glutathione; GPxs: Glutathione peroxidase; HO·: Hydroxyl radicals; Interferon-γ: IFN-γ; IRF3: interferon regulatory factor 3; IRF7: interferon regulatory factor 7; ISRE: Interferon-Sensitive Response Elements; KEAP1:: Kelch-like ECH-associated protein 1; MAVS: mitochondrial antiviral signaling protein; MSK1: Mitogen- and Stress-Activated Kinase 1; MitoQ: mitoquidone mesylate; MTOC: mitochondrial microtubule-organizing center; NAC: N-acetyl-L-cysteine; NADPH: Nicotinamide adenine dinucleotide phosphate; NETs: Neutrophil Extracellular Traps; NF-κB: nuclear factor kappa-light-chain-enhancer of activated B cells; NRF2: Nuclear factor-erythroid 2 related factor2; NLRP3 inflammasome: Nod-like receptor protein3 inflammasome; NOX: NADPH oxidases; NRF2: Nuclear factor erythroid 2-related factor 2; PML-NBs; promyelocytic leukemia protein- nuclear bodies; RIG-I: Retinoic acid-inducible gene-I; RNF4-PML (RING finger protein 4-promyelocytic leukemia; SOD: Superoxide dismutase; siRNAs: short interfering RNAs; TLR: Toll like receptor; UPR: Unfolded protein response.

## References

[1] Li Y., Wang X., Blau D.M., Caballero M.T., Feikin D.R., Gill C.J., Madhi S.A., Omer S.B., Simões E.A.F., Campbell H. (2022). Global, regional, and national disease burden estimates of acute lower respiratory infections due to respiratory syncytial virus in children younger than 5 years in 2019: A systematic analysis. Lancet.

[2] Mazur N.I., Caballero M.T., Nunes M.C. (2024). Severe respiratory syncytial virus infection in children: Burden, management, and emerging therapies. Lancet.

[3] Larrañaga C.L., Ampuero S.L., Luchsinger V.F., Carrión F.A., Aguilar N.V., Morales P.R., Palomino M.A., Tapia L.F., Avendaño L.F. (2009). Impaired immune response in severe human lower tract respiratory infection by respiratory syncytial virus. Pediatr. Infect. Dis. J..

[4] Mella C., Suarez-Arrabal M.C., Lopez S., Stephens J., Fernandez S., Hall M.W., Ramilo O., Mejias A. (2013). Innate immune dysfunction is associated with enhanced disease severity in infants with severe respiratory syncytial virus bronchiolitis. J. Infect. Dis..

[5] Bergeron H.C., Tripp R.A. (2021). Immunopathology of RSV: An Updated Review. Viruses.

[6] Bai G., Huang X., He Y. (2026). Advances in respiratory syncytial virus therapeutics: A comprehensive review of small-molecule inhibitors, monoclonal antibodies, and vaccines. Eur. J. Med. Chem..

[7] Aherne W., Bird T., Court S.D., Gardner P.S., McQuillin J. (1970). Pathological changes in virus infections of the lower respiratory tract in children. J. Clin. Pathol..

[8] Johnson J.E., Gonzales R.A., Olson S.J., Wright P.F., Graham B.S. (2007). The histopathology of fatal untreated human respiratory syncytial virus infection. Mod. Pathol..

[9] Villenave R., Shields M.D., Power U.F. (2013). Respiratory syncytial virus interaction with human airway epithelium. Trends Microbiol..

[10] Rossi G.A., Colin A.A. (2017). Respiratory syncytial virus-Host interaction in the pathogenesis of bronchiolitis and its impact on respiratory morbidity in later life. Pediatr. Allergy Immunol..

[11] Garofalo R.P., Kolli D., Casola A. (2013). Respiratory syncytial virus infection: Mechanisms of redox control and novel therapeutic opportunities. Antioxid. Redox Signal..

[12] Mittal M., Siddiqui M.R., Tran K., Reddy S.P., Malik A.B. (2014). Reactive oxygen species in inflammation and tissue injury. Antioxid. Redox Signal..

[13] Yang X., Liu X., Nie Y., Zhan F., Zhu B. (2023). Oxidative stress and ROS-mediated cellular events in RSV infection: Potential protective roles of antioxidants. Virol. J..

[14] Peterhans E. (1997). Oxidants and antioxidants in viral diseases: Disease mechanisms and metabolic regulation. J. Nutr..

[15] Komaravelli N., Tian B., Ivanciuc T., Mautemps N., Brasier A.R., Garofalo R.P., Casola A. (2015). Respiratory syncytial virus infection down-regulates antioxidant enzyme expression by triggering deacetylation-proteasomal degradation of Nrf2. Free Radic. Biol. Med..

[16] Averill-Bates D. (2024). Reactive oxygen species and cell signaling. Review. Biochim. Biophys. Acta Mol. Cell Res..

[17] Knaus U.G. (2021). Oxidants in Physiological Processes. Handb. Exp. Pharmacol..

[18] Kowaltowski A.J., de Souza-Pinto N.C., Castilho R.F., Vercesi A.E. (2009). Mitochondria and reactive oxygen species. Free Radic. Biol. Med..

[19] Covarrubias A.J., Perrone R., Grozio A., Verdin E. (2021). NAD^+^ metabolism and its roles in cellular processes during ageing. Nat. Rev. Mol. Cell Biol..

[20] Crane F.L., Low H. (2008). Reactive oxygen species generation at the plasma membrane for antibody control. Autoimmun. Rev..

[21] Thomas C., Mackey M.M., Diaz A.A., Cox D.P. (2009). Hydroxyl radical is produced via the Fenton reaction in submitochondrial particles under oxidative stress: Implications for diseases associated with iron accumulation. Redox Rep..

[22] Snezhkina A.V., Kudryavtseva A.V., Kardymon O.L., Savvateeva M.V., Melnikova N.V., Krasnov G.S., Dmitriev A.A. (2019). ROS Generation and Antioxidant Defense Systems in Normal and Malignant Cells. Oxid. Med. Cell Longev..

[23] Cao S.S., Kaufman R.J. (2014). Endoplasmic reticulum stress and oxidative stress in cell fate decision and human disease. Antioxid. Redox Signal..

[24] Valko M., Leibfritz D., Moncol J., Cronin M.T., Mazur M., Telser J. (2007). Free radicals and antioxidants in normal physiological functions and human disease. Int. J. Biochem. Cell Biol..

[25] Hajam Y.A., Rani R., Ganie S.Y., Sheikh T.A., Javaid D., Qadri S.S., Pramodh S., Alsulimani A., Alkhanani M.F., Harakeh S. (2022). Oxidative Stress in Human Pathology and Aging: Molecular Mechanisms and Perspectives. Cells.

[26] Foo J., Bellot G., Pervaiz S., Alonso S. (2022). Mitochondria-mediated oxidative stress during viral infection. Trends Microbiol..

[27] Andrieux P., Chevillard C., Cunha-Neto E., Nunes J.P.S. (2021). Mitochondria as a Cellular Hub in Infection and Inflammation. Int. J. Mol. Sci..

[28] Lee C. (2018). Therapeutic Modulation of Virus-Induced Oxidative Stress via the Nrf2-Dependent Antioxidative Pathway. Oxid. Med. Cell Longev..

[29] Daskou M., Fotooh Abadi L., Gain C., Wong M., Sharma E., Kombe Kombe A.J., Nanduri R., Kelesidis T. (2023). The Role of the NRF2 Pathway in the Pathogenesis of Viral Respiratory Infections. Pathogens.

[30] Kombe Kombe A.J., Fotoohabadi L., Nanduri R., Gerasimova Y., Daskou M., Gain C., Sharma E., Wong M., Kelesidis T. (2024). The Role of the Nrf2 Pathway in Airway Tissue Damage Due to Viral Respiratory Infections. Int. J. Mol. Sci..

[31] Hosakote Y.M., Jantzi P.D., Esham D.L., Spratt H., Kurosky A., Casola A., Garofalo R.P. (2011). Viral-mediated inhibition of antioxidant enzymes contributes to the pathogenesis of severe respiratory syncytial virus bronchiolitis. Am. J. Respir. Crit. Care Med..

[32] Onomoto K., Onoguchi K., Yoneyama M. (2021). Regulation of RIG-I-like receptor-mediated signaling: Interaction between host and viral factors. Cell. Mol. Immunol..

[33] Thoresen D.T., Galls D., Götte B., Wang W., Pyle A.M. (2023). A rapid RIG-I signaling relay mediates efficient antiviral response. Mol. Cell.

[34] Hu M., Schulze K.E., Ghildyal R., Henstridge D.C., Kolanowski J.L., New E.J., Hong Y., Hsu A.C., Hansbro P.M., Wark P.A. (2019). Respiratory syncytial virus co-opts host mitochondrial function to favour infectious virus production. eLife.

[35] Jamaluddin M., Tian B., Boldogh I., Garofalo R.P., Brasier A.R. (2009). Respiratory syncytial virus infection induces a reactive oxygen species-MSK1-phospho-Ser-276 RelA pathway required for cytokine expression. J. Virol..

[36] Bhoj V.G., Sun Q., Bhoj E.J., Somers C., Chen X., Torres J.P., Mejias A., Gomez A.M., Jafri H., Ramilo O. (2008). MAVS and MyD88 are essential for innate immunity but not cytotoxic T lymphocyte response against respiratory syncytial virus. Proc. Natl. Acad. Sci. USA.

[37] Casola A., Burger N., Liu T., Jamaluddin M., Brasier A.R., Garofalo R.P. (2001). Oxidant tone regulates RANTES gene expression in airway epithelial cells infected with respiratory syncytial virus. Role in viral-induced interferon regulatory factor activation. J. Biol. Chem..

[38] Liu P., Jamaluddin M., Li K., Garofalo R.P., Casola A., Brasier A.R. (2007). Retinoic acid-inducible gene I mediates early antiviral response and toll-like receptor 3 expression in respiratory syncytial virus-infected airway epithelial cells. J. Virol..

[39] Cervantes-Ortiz S.L., Zamorano Cuervo N., Grandvaux N. (2016). Respiratory syncytial virus and cellular stress responses: Impact on replication and physiopathology. Viruses.

[40] Walter P., Ron D. (2011). The unfolded protein response: From stress pathway to homeostatic regulation. Science.

[41] Kirsebom F.C.M., Kausar F., Nuriev R., Makris S., Johansson C. (2019). Neutrophil recruitment and activation are differentially dependent on MyD88/TRIF and MAVS signaling during RSV infection. Mucosal Immunol..

[42] Bataki E.L., Evans G.S., Everard M.L. (2005). Respiratory syncytial virus and neutrophil activation. Clin. Exp. Immunol..

[43] Hosakote Y.M., Liu T., Castro S.M., Garofalo R.P., Casola A. (2009). Respiratory syncytial virus induces oxidative stress by modulating antioxidant enzymes. Am. J. Respir. Cell Mol. Biol..

[44] Cho H.Y., Imani F., Miller-DeGraff L., Walters D., Melendi G.A., Yamamoto M., Polack F.P., Kleeberger S.R. (2009). Antiviral activity of Nrf2 in a murine model of respiratory syncytial virus disease. Am. J. Respir. Crit. Care Med..

[45] Habibi M.S., Thwaites R.S., Chang M., Jozwik A., Paras A., Kirsebom F., Varese A., Owen A., Cuthbertson L., James P. (2020). Neutrophilic inflammation in the respiratory mucosa predisposes to RSV infection. Science.

[46] Fuchs T.A., Abed U., Goosmann C., Hurwitz R., Schulze I., Wahn V., Weinrauch Y., Brinkmann V., Zychlinsky A. (2007). Novel cell death program leads to neutrophil extracellular traps. J. Cell Biol..

[47] Wang Y., Du C., Zhang Y., Zhu L. (2024). Composition and Function of Neutrophil Extracellular Traps. Biomolecules.

[48] Papayannopoulos V. (2018). Neutrophil extracellular traps in immunity and disease. Nat. Rev. Immunol..

[49] Muraro S.P., De Souza G.F., Gallo S.W., Da Silva B.K., De Oliveira S.D., Vinolo M.A.R., Saraiva E.M., Porto B.N. (2018). Respiratory Syncytial Virus induces the classical ROS-dependent NETosis through PAD-4 and necroptosis pathways activation. Sci. Rep..

[50] Souza P.S.S., Barbosa L.V., Diniz L.F.A., da Silva G.S., Lopes B.R.P., Souza P.M.R., de Araujo G.C., Pessoa D., de Oliveira J., Souza F.P. (2018). Neutrophil extracellular traps possess anti-human respiratory syncytial virus activity: Possible interaction with the viral F protein. Virus Res..

[51] Diniz L.F.A., Matsuba B.K., Souza P.S.S., Lopes B.R.P., Kubo L.H., Oliveira J., Toledo K.A. (2021). Effects of neutrophil extracellular traps during human respiratory syncytial virus infection in vitro. Braz. J. Biol..

[52] Sebina I., Rashid R.B., Sikder M.A.A., Rahman M.M., Ahmed T., Radford-Smith D.E., Kotenko S.V., Hill G.R., Bald T., Phipps S. (2022). IFN-λ Diminishes the Severity of Viral Bronchiolitis in Neonatal Mice by Limiting NADPH Oxidase-Induced PAD4-Independent NETosis. J. Immunol..

[53] Cortjens B., de Boer O.J., de Jong R., Antonis A.F., Sabogal Piñeros Y.S., Lutter R., van Woensel J.B., Bem R.A. (2016). Neutrophil extracellular traps cause airway obstruction during respiratory syncytial virus disease. J. Pathol..

[54] Deretic V., Saitoh T., Akira S. (2013). Autophagy in infection, inflammation and immunity. Nat. Rev. Immunol..

[55] Chen L., Yang L., Li Y., Liu T., Yang B., Liu L., Wu R. (2023). Autophagy and Inflammation: Regulatory Roles in Viral Infections. Biomolecules.

[56] Aligolighasemabadi F., Bakinowska E., Kiełbowski K., Sadeghdoust M., Coombs K.M., Mehrbod P., Ghavami S. (2025). Autophagy and Respiratory Viruses: Mechanisms, Viral Exploitation, and Therapeutic Insights. Cells.

[57] Jackson W.T., Giddings T.H., Taylor M.P., Mulinyawe S., Rabinovitch M., Kopito R.R., Kirkegaard K. (2005). Subversion of cellular autophagosomal machinery by RNA viruses. PLoS Biol..

[58] Morris S., Swanson M.S., Lieberman A., Reed M., Yue Z., Lindell D.M., Lukacs N.W. (2011). Autophagy-mediated dendritic cell activation is essential for innate cytokine production and APC function with respiratory syncytial virus responses. J. Immunol..

[59] Reed M., Morris S.H., Jang S., Mukherjee S., Yue Z., Lukacs N.W. (2013). Autophagy-inducing protein beclin-1 in dendritic cells regulates CD4 T cell responses and disease severity during respiratory syncytial virus infection. J. Immunol..

[60] Li M., Li J., Zeng R., Yang J., Liu J., Zhang Z., Song X., Yao Z., Ma C., Li W. (2018). Respiratory Syncytial Virus Replication Is Promoted by Autophagy-Mediated Inhibition of Apoptosis. J. Virol..

[61] Thorburn A. (2008). Apoptosis and autophagy: Regulatory connections between two supposedly different processes. Apoptosis.

[62] Bitko V., Shulyayeva O., Mazumder B., Musiyenko A., Ramaswamy M., Look D.C., Barik S. (2007). Nonstructural proteins of respiratory syncytial virus suppress premature apoptosis by an NF-kappaB-dependent, interferon-independent mechanism and facilitate virus growth. J. Virol..

[63] Han B., Wang Y., Zheng M. (2021). Inhibition of autophagy promotes human RSV NS1-induced inflammation and apoptosis in vitro. Exp. Ther. Med..

[64] Fu J., Wu H. (2023). Structural Mechanisms of NLRP3 Inflammasome Assembly and Activation. Annu. Rev. Immunol..

[65] Bai Y., Pan Y., Liu X. (2025). Mechanistic insights into gasdermin-mediated pyroptosis. Nat. Rev. Mol. Cell Biol..

[66] Zhao C., Zhao W. (2020). NLRP3 Inflammasome—A Key Player in Antiviral Responses. Front. Immunol..

[67] Lin R., Porto B.N. (2025). Pyroptosis in Respiratory Virus Infections: A Narrative Review of Mechanisms, Pathophysiology, and Potential Therapeutic Interventions. Microorganisms.

[68] Segovia J., Sabbah A., Mgbemena V., Tsai S.Y., Chang T.H., Berton M.T., Morris I.R., Allen I.C., Ting J.P., Bose S. (2012). TLR2/MyD88/NF-κB pathway, reactive oxygen species, potassium efflux activates NLRP3/ASC inflammasome during respiratory syncytial virus infection. PLoS ONE.

[69] Takeuchi R., Tsutsumi H., Osaki M., Sone S., Imai S., Chiba S. (1998). Respiratory syncytial virus infection of neonatal monocytes stimulates synthesis of interferon regulatory factor 1 and interleukin-1beta (IL-1beta)-converting enzyme and secretion of IL-1beta. J. Virol..

[70] Schuler C.F., Malinczak C.A., Best S.K.K., Morris S.B., Rasky A.J., Ptaschinski C., Lukacs N.W., Fonseca W. (2020). Inhibition of uric acid or IL-1β ameliorates respiratory syncytial virus immunopathology and development of asthma. Allergy.

[71] Vu L.D., Phan A.T.Q., Hijano D.R., Siefker D.T., Tillman H., Cormier S.A. (2022). IL-1β Promotes Expansion of IL-33^+^ Lung Epithelial Stem Cells after Respiratory Syncytial Virus Infection during Infancy. Am. J. Respir. Cell Mol. Biol..

[72] Lee S.A., Kwak M.S., Kim S., Shin J.S. (2014). The role of high mobility group box 1 in innate immunity. Yonsei Med. J..

[73] Bianchi M.E., Crippa M.P., Manfredi A.A., Mezzapelle R., Rovere Querini P., Venereau E. (2017). High-mobility group box 1 protein orchestrates responses to tissue damage via inflammation, innate and adaptive immunity, and tissue repair. Immunol. Rev..

[74] Gaboriaud C., Lorvellec M., Rossi V., Dumestre-Pérard C., Thielens N.M. (2022). Complement System and Alarmin HMGB1 Crosstalk: For Better or Worse. Front. Immunol..

[75] Yu Y., Tang D., Kang R. (2015). Oxidative stress-mediated HMGB1 biology. Front. Physiol..

[76] Manti S., Harford T.J., Salpietro C., Rezaee F., Piedimonte G. (2018). Induction of high-mobility group Box-1 in vitro and in vivo by respiratory syncytial virus. Pediatr. Res..

[77] Loh Z., Simpson J., Ullah A., Zhang V., Gan W.J., Lynch J.P., Werder R.B., Sikder A.A., Lane K., Sim C.B. (2020). HMGB1 amplifies ILC2-induced type-2 inflammation and airway smooth muscle remodelling. PLoS Pathog..

[78] Chen S., Yu G., Xie J., Tang W., Gao L., Long X., Ren L., Xie X., Deng Y., Fu Z. (2019). High-mobility group box-1 protein from CC10^+^ club cells promotes type 2 response in the later stage of respiratory syncytial virus infection. Am. J. Physiol. Lung Cell. Mol. Physiol..

[79] Fonseca W., Lukacs N.W., Elesela S., Malinczak C.A. (2021). Role of ILC2 in Viral-Induced Lung Pathogenesis. Front. Immunol..

[80] Rossi G.A., Ballarini S., Salvati P., Sacco O., Colin A.A. (2022). Alarmins and innate lymphoid cells 2 activation: A common pathogenetic link connecting respiratory syncytial virus bronchiolitis and later wheezing/asthma?. Pediatr. Allergy Immunol..

[81] Norlander A.E., Peebles R.S. (2020). Innate Type 2 Responses to Respiratory Syncytial Virus Infection. Viruses.

[82] Legg J.P., Hussain I.R., Warner J.A., Johnston S.L., Warner J.O. (2003). Type 1 and type 2 cytokine imbalance in acute respiratory syncytial virus bronchiolitis. Am. J. Respir. Crit. Care Med..

[83] Bui R.H., Molinaro G.A., Kettering J.D., Heiner D.C., Imagawa D.T., St Geme J.W. (1987). Virus-specific IgE and IgG4 antibodies in serum of children infected with respiratory syncytial virus. J. Pediatr..

[84] Welliver R.C., Wong D.T., Sun M., Middleton E., Vaughan R.S., Ogra P.L. (1981). The development of respiratory syncytial virus-specific IgE and the release of histamine in nasopharyngeal secretions after infection. N. Engl. J. Med..

[85] Welliver R.C., Sun M., Rinaldo D., Ogra P.L. (1986). Predictive value of respiratory syncytial virus-specific IgE responses for recurrent wheezing following bronchiolitis. J. Pediatr..

[86] Openshaw P.J., Dean G.S., Culley F.J. (2003). Links between respiratory syncytial virus bronchiolitis and childhood asthma: Clinical and research approaches. Pediatr. Infect. Dis. J..

[87] Castro S.M., Guerrero-Plata A., Suarez-Real G., Adegboyega P.A., Colasurdo G.N., Khan A.M., Garofalo R.P., Casola A. (2006). Antioxidant treatment ameliorates respiratory syncytial virus-induced disease and lung inflammation. Am. J. Respir. Crit. Care Med..

[88] Wyde P.R., Moore D.K., Pimentel D.M., Gilbert B.E., Nimrod R., Panet A. (1996). Recombinant superoxide dismutase (SOD) administered by aerosol inhibits respiratory syncytial virus infection in cotton rats. Antivir. Res..

[89] Chi L., Shan Y., Cui Z. (2022). N-Acetyl-L-Cysteine Protects Airway Epithelial Cells during Respiratory Syncytial Virus Infection against Mucin Synthesis, Oxidative Stress, and Inflammatory Response and Inhibits HSPA6 Expression. Anal. Cell. Pathol..

[90] Silvestri M., Rossi G.A. (2013). Melatonin: Its possible role in the management of viral infections—A brief review. Ital. J. Pediatr..

[91] Huang S.H., Cao X.J., Liu W., Shi X.Y., Wei W. (2010). Inhibitory effect of melatonin on lung oxidative stress induced by respiratory syncytial virus infection in mice. J. Pineal Res..

[92] Rossi G.A., Sacco O., Capizzi A., Mastromarino P. (2021). Can Resveratrol-Inhaled Formulations Be Considered Potential Adjunct Treatments for COVID-19?. Front. Immunol..

[93] Bohara R.A., Tabassum N., Singh M.P., Gigli G., Ragusa A., Leporatti S. (2022). Recent Overview of Resveratrol’s Beneficial Effects and Its Nano-Delivery Systems. Molecules.

[94] Adams T.J., Schuliga M., Pearce N., Bartlett N.W., Liang M. (2025). Targeting respiratory virus-induced reactive oxygen species in airways diseases. Eur. Respir. Rev..

[95] Eisenreich W., Rudel T., Heesemann J., Goebel W. (2019). How viral and intracellular bacterial pathogens reprogram the metabolism of host cells to allow their intracellular replication. Front. Cell. Infect. Microbiol..

[96] Barkauskas C.E., Chung M.I., Fioret B., Gao X., Katsura H., Hogan B.L. (2017). Lung organoids: Current uses and future promise. Development.

[97] Joo H., Min S., Cho S.W. (2024). Advanced lung organoids for respiratory system and pulmonary disease modeling. J. Tissue Eng..

[98] Mao X., Cai M., Fan B. (2026). Lung organoids for respiratory diseases: Overcoming translational hurdles in drug discovery and safety assessment. Front. Bioeng. Biotechnol..

